# Evaluation of a Semiautomated System for the Quantitation of Human Adenovirus DNA from Clinical Samples

**DOI:** 10.1128/spectrum.05010-22

**Published:** 2023-02-27

**Authors:** Jordan Mah, Chun Hong Huang, Malaya K. Sahoo, Benjamin A. Pinsky

**Affiliations:** a Department of Pathology, Stanford University School of Medicine, Stanford, California, USA; b Department of Medicine, Division of Infectious Diseases and Geographic Medicine, Stanford University School of Medicine, Stanford, California, USA; Quest Diagnostics

**Keywords:** adenoviruses, automation, bone marrow transplantation, diagnostics, immunocompromised hosts, infectious disease, transplant infectious diseases, virology

## Abstract

Human adenoviruses (HAdVs) cause severe disease in immunocompromised patients. Quantitation of HAdV DNA in peripheral blood is used to assess the risk of disseminated disease and to monitor response to therapy. The lower limit of detection, precision, and linearity of the semiautomated AltoStar adenovirus quantitative PCR (qPCR) was evaluated using reference HAdV-E4 in EDTA plasma and respiratory virus matrix. Qualitative and quantitative agreement was determined using 122 clinical EDTA plasma specimens previously tested using a laboratory-developed HAdV qPCR. The 95% lower limit of detection (LLOD) was 33 IU/mL (95% confidence interval [CI], 10 to 56) for EDTA plasma and 188 IU/mL (95% CI, 145 to 304) for respiratory swab matrix. In both matrices, the AltoStar HAdV qPCR was linear from 7.0 to 2.0 log_10_ IU/mL. For the clinical specimens, overall agreement was 96.7% (95% CI, 91.8 to 99.1), positive percent agreement was 95.5% (95% CI, 87.6 to 98.5), and negative percent agreement was 98.2% (95% CI, 88.5 to 99.7). Passing-Bablok analysis of specimens quantifiable by both methods revealed a regression line of Y = 1.11 · X + 0.00; there was positive proportional bias (95% CI of the slope, 1.05 to 1.22) but no systematic bias (95% CI of the Y-intercept, −0.43 to 0.23) compared to the reference. The AltoStar platform provides accurate quantitation of HAdV DNA and provides a semiautomated option for the clinical monitoring of HAdV following transplantation.

**IMPORTANCE** Accurate quantification of human adenovirus DNA in the peripheral blood plays a critical role in the management of adenovirus infections in transplant recipients. Many laboratories utilize in-house laboratory-based PCR assays for the quantification of human adenovirus, as there are few commercial options available. Here, we describe the analytical and clinical performance of the semiautomated AltoStar adenovirus quantitative PCR (Altona Diagnostics). This platform provides sensitive, precise, and accurate quantification of adenovirus DNA that is well suited for virological testing following transplantation. Prior to implementing a new quantitative test in the clinical laboratory, a rigorous evaluation is required to determine assay performance characteristics and to correlate results to current in-house methods of quantitation.

## INTRODUCTION

Human adenoviruses (HAdVs) are nonenveloped, double-stranded DNA viruses in the genus *Mastadenovirus*, comprising eight species (A to G) and at least 111 genotypes (1, 2). These viruses most commonly cause self-limited respiratory tract infections; however, they also cause gastrointestinal, genitourinary, and ophthalmologic disease in immunocompetent children and adults (3). HAdVs are particularly important in immunocompromised patients, including both solid organ and hematopoietic cell transplant (HCT) recipients, where infection may result in graft failure or severe disseminated disease (4).

Diagnosis of HAdV infection relies on detection of viral genomic DNA by nucleic acid amplification tests. For example, HAdV is typically included as a qualitative target on expanded upper respiratory virus panels (5). Quantitative PCR (qPCR) also plays an important role in the diagnosis and management of HAdV in the immunocompromised host. Given the myriad clinical manifestations of HAdV infection, serial monitoring of the peripheral blood by HAdV qPCR may be indicated for immunocompromised patients that present with a compatible syndrome (3, 4). HAdV qPCR screening of the peripheral blood of asymptomatic pediatric allogeneic HCT recipients is also commonly performed to inform preemptive therapy (6, 7). Finally, HAdV DNA levels in the peripheral blood may be utilized to monitor the response to antiviral therapy, as well as to guide changes in immunosuppression (3, 4).

Given the relatively limited number of commercial options for quantitative HAdV testing, we sought to evaluate the semiautomated AltoStar adenovirus PCR (Altona Diagnostics) using HAdV reference panels in both plasma and respiratory swab matrix, as well as clinical plasma specimens.

## RESULTS

### AltoStar HAdV analytical performance.

The 95% lower limit of detection (LLOD) was 33 IU/mL (95% confidence interval [CI], 10 to 56) for EDTA plasma and 188 IU/mL (95% CI, 145 to 304) for respiratory swab matrix. Within-run (repeatability), between-run (reproducibility), and within-laboratory (total) imprecision at low levels of HAdV DNA are described for EDTA plasma (Table 1) and respiratory swab matrix (Table 2). As expected from the 95% LLOD values, imprecision could be calculated as low as 75 IU/mL for EDTA plasma and 250 IU/mL for respiratory swab matrix.

**TABLE 1:** Imprecision of the AltoStar HAdV qPCR at low concentrations of HAdV DNA in EDTA plasma^*a*^

Nominal concn (IU/mL)	Nominal concn (log_10_ IU/mL)	Observed mean (log_10_ copies/mL)	SD within-run	% CV within-run	SD between-run	% CV between-run	SD total	% CV total
1,000	3.00	3.69	0.05	1.47	0.00	0.03	0.05	1.37
750	2.88	3.59	0.08	2.26	0.05	1.33	0.09	2.50
500	2.70	3.48	0.14	3.90	0.01	0.38	0.13	3.67
250	2.40	3.16	0.12	3.84	0.05	1.45	0.12	3.88
125	2.10	2.88	0.20	6.82	0.02	0.73	0.18	6.42
75	1.88	2.55	0.32	12.44	0.03	1.01	0.30	11.68

a CV, coefficient of variation.

**TABLE 2:** Imprecision of the AltoStar HAdV qPCR at low concentrations of HAdV DNA in respiratory swab matrix^*a*^

Nominal concn (IU/mL)	Nominal concn (log_10_ IU/mL)	Observed mean (log_10_ copies/mL)	SD within-run	% CV within-run	SD between-run	% CV between-run	SD total	% CV total
1,000	3.00	2.93	0.16	5.47	0.11	3.60	0.18	6.25
750	2.88	2.74	0.16	6.00	0.14	5.10	0.21	7.58
500	2.70	2.50	0.20	8.00	0.11	4.21	0.21	8.58
250	2.40	2.13	0.25	11.55	0.11	5.06	0.25	11.93

a CV, coefficient of variation.

**FIG 1:**
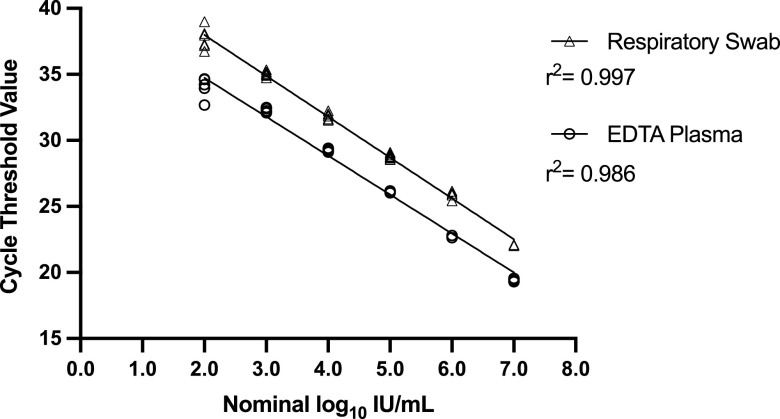
Linear regression of AltoStar cycle threshold values using 10-fold dilutions of HAdV-E4 in EDTA plasma (open circles) and respiratory swab matrix (open triangles).

To assess the linearity for both matrices, the observed cycle threshold (*C_T_*) values were plotted against the nominal values, and ordinary least-squares regression was performed (Fig. 1). This analysis revealed that the AltoStar HAdV qPCR is linear from 7.0 to 2.0 log_10_ IU/mL in both EDTA plasma and respiratory swab matrix.

### Evaluation of the AltoStar HAdV assay using clinical plasma samples.

A total of 122 EDTA plasma samples (65 detected, 57 not detected) originally tested using a laboratory-developed HAdV qPCR were tested using the AltoStar HAdV qPCR. Using the laboratory-developed test as a reference (Table 3), overall agreement was 96.7% (95% CI, 91.8 to 99.1), positive percent agreement was 95.5% (95% CI, 87.6 to 98.5), and negative percent agreement was 98.2% (95% CI, 88.5 to 99.7). The kappa coefficient was 0.93 (95% CI, 0.87 to 1.0).

**TABLE 3:** Qualitative agreement of AltoStar HAdV with reference HAdV qPCR testing^*a*^

		Reference		Positive percent agreement (95% CI)	Negative percent agreement (95% CI)
		Detected	Not detected		
AltoStar	Detected	64	3	95.5% (87.6–98.5)	98.2 (88.5–99.7)
	Not Detected	1	54

a CI, confidence interval.

There were a total of four discrepant samples. One sample was detected by the reference but not detected by AltoStar. This sample was originally quantitated at 2.56 log_10_ copies/mL. However, HAdV DNA was not detected by the reference when qPCR testing was performed from a newly extracted eluate. Three samples were detected by AltoStar but not detected by the reference qPCR. These samples were quantitated at 1.92, 1.89, and 1.59 log_10_ copies/mL. When reference testing was performed from a newly extracted eluate, all three samples remained negative.

**FIG 2:**
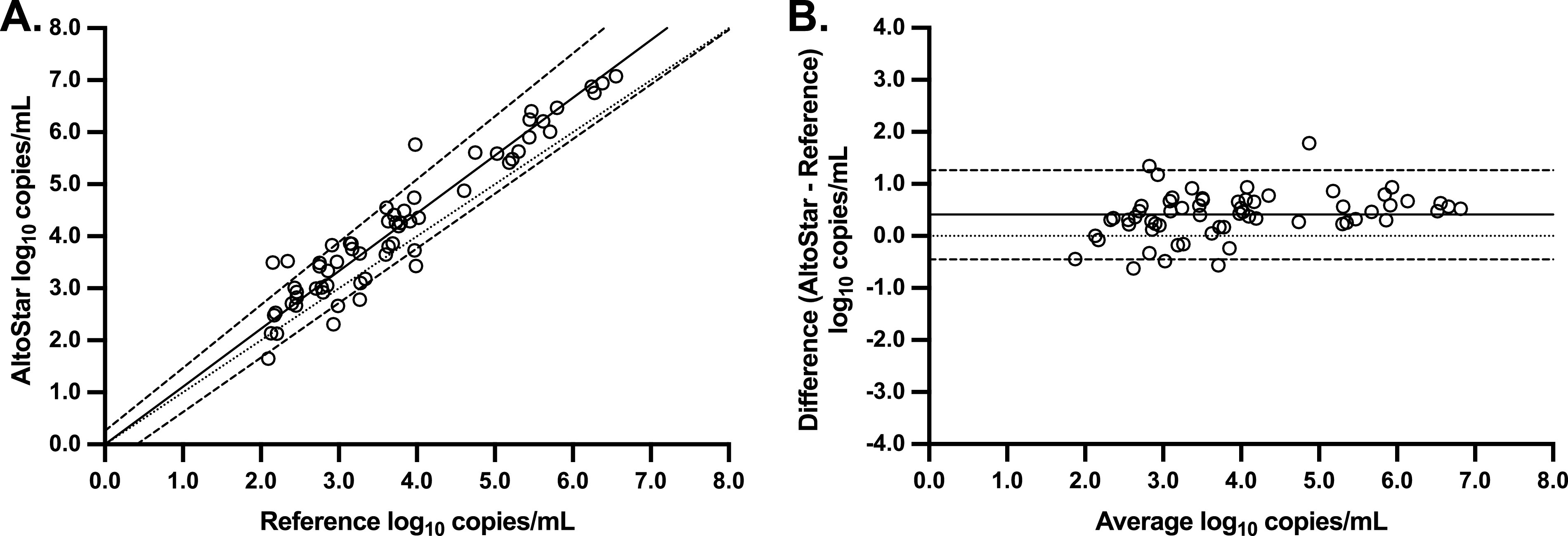
(A and B) Passing-Bablok regression (A) and Bland-Altman analysis (B) comparing adenovirus DNA levels (log_10_ copies/mL) between AltoStar and reference qPCR methods in EDTA plasma. Passing-Bablok: solid line, regression line; dotted line, line of identity; dashed lines, 95% confidence intervals. Bland-Altman: solid line, mean difference; dotted line, zero difference; dashed lines, 95% confidence intervals.

To investigate the quantitative agreement between AltoStar HAdV qPCR and the reference, the log_10_ copies/mL of the 62 clinical samples quantifiable by both methods were plotted against one another, and Passing-Bablok regression was performed (Fig. 2A). This analysis resulted in a regression line of Y = 1.11 · X + 0.00. The 95% confidence interval of the slope (1.05 to 1.22) indicates a positive proportional bias compared to the reference. However, no systematic bias was observed, as the 95% confidence interval of the Y-intercept (−0.43 to 0.23) includes zero. Next, the differences in log_10_ copies/mL were plotted against the average values to generate a Bland-Altman plot (Fig. 2B). This analysis revealed a bias of 0.41 log_10_ copies/mL (AltoStar – reference HAdV PCR) and 95% limits of agreement of −0.45 to 1.26.

## DISCUSSION

In this study we describe the analytical and clinical performance characteristics of the semiautomated AltoStar adenovirus PCR. In the analytical phase, we evaluated the two most important clinical matrices, EDTA plasma and respiratory swab matrix, and demonstrated that the LLOD, precision, and linearity met requirements for clinical use. The plasma results verified the analytical performance reported in the AltoStar adenovirus PCR CE *in vitro* diagnostic (IVD) package insert (8) and were similar to previously published evaluations of the nonautomated Altona RealStar adenovirus reagents (9, 10). Comparison of the AltoStar with a laboratory-developed reference qPCR using clinical EDTA plasma samples demonstrated qualitative agreement and slight positive proportional bias.

Though quantitative testing is frequently used for clinical decision-making, thresholds for initiating preemptive antiviral therapy vary widely across institutions (from 2.0 to 6.0 log_10_ copies/mL) (6, 7). This variation is likely due, in part, to the use of different calibrators. The availability of the 1st WHO International Standard for human adenovirus DNA for nucleic acid amplification techniques (National Institute for Biological Standards and Controls, code 16/324) provides the opportunity for harmonization, but additional studies will be required to ensure commutability and, ultimately, overall improvement in quantitative agreement. Nevertheless, the qPCRs evaluated in this study showed good quantitative agreement despite the use of different calibrators. In the absence of a universal HAdV threshold, monitoring viral DNA kinetics in the peripheral blood may help predict the development of HAdV disease and remains an important tool to confirm response to therapy (3, 4, 11).

The strengths of this study include the number of replicates used in the analytical evaluation and the robust number of clinical samples used in the comparison of qPCR methods. Though not a fully automated, sample-to-answer system, the AltoStar AM16 automates extraction and assay set-up and allows up to four qPCR assays to be performed from a single specimen. Given the shared cycling parameters across the HAdV assay and other AltoStar transplant virus qPCRs (cytomegalovirus, Epstein-Barr virus, BK virus, etc.), this system may be particularly well suited for virologic testing following transplantation.

The limitations of this study include the lack of harmonization to the WHO international standard, as discussed above, and the absence of HAdV typing data for the clinical samples used in the method comparison. Though previously published evaluations of the nonautomated Altona RealStar adenovirus reagents demonstrate similar performance across types (9, 10), future work will be required to verify accurate type-specific quantitation with the AltoStar HAdV qPCR.

In summary, the semiautomated AltoStar platform provides highly sensitive, precise, and accurate quantitation of HAdV DNA in clinical specimens. Detailed assay evaluation is required when implementing new HAdV qPCR tests in the clinical virology laboratory.

## MATERIALS AND METHODS

### Ethics statement.

This study was conducted with Stanford institutional review board approval (protocol 46794), and individual consent was waived.

### Human adenovirus PCR on the AltoStar AM16.

The AltoStar AM16 (Altona Diagnostics) is a robotic liquid handler for automated nucleic acid extraction and PCR setup. For each run, a .psv file containing specimen and assay information was uploaded to the AM16 AltoStar Connect software version 1.7.6, which guides the loading of extraction reagents, tips, plates, and other consumables, as well as barcoded specimen tubes. The software also ensures that reagent and specimen volumes are sufficient and that reagent lots are not mixed.

For each specimen, 500 μL was processed using the AltoStar purification kit version 1.5, and the extracted nucleic acids were eluted in 80 μL. Each extraction included a negative control (defibrinated human plasma; SeraCare Life Sciences), and HAdV low positive and high positive controls (Exact Diagnostics). The AltoStar AM16 adds internal-control nucleic acids to the lysed primary sample. The PCR was set up after loading the required set of AltoStar adenovirus PCR kit version 1.0 reagents and consumables, as guided by the software interface. Each PCR consisted of 18 μL general PCR reagent (GPR), 1 μL primer mix, 1 μL probe mix, and 10 μL eluate. Thermal cycling was performed on a Bio-Rad CFX96 real-time PCR instrument using the following conditions: 95°C for 10 min, and then 45 cycles of 95°C for 15 s and 58.0°C for 1 min. Each PCR plate contained a no-template control (nuclease-free water; New England Biolabs) and a 4-point standard curve in copies/mL. Fluorescence was collected in the Altona_FAM (HAdV) and Altona_JOE (internal control) channels, and thresholds were set at 1,000 and 500 relative fluorescence units (RFU), respectively. Sample information was compiled in the plate file generated by AltoStar Connect and was imported into Bio-Rad CFX Maestro software.

### Analytical evaluation.

Custom panels comprising HAdV-E4 were manufactured by Exact Diagnostics to determine the 95% lower limit of detection (LLOD), precision at low levels of viral DNA, and assay linearity. The LLOD panels consisted of EDTA plasma or upper respiratory swab specimen matrix at 1,000, 750, 500, 250, 125, 75, and 10 IU/mL. A total of 16 replicates were tested at each level; 8 replicates were tested per day on 2 separate days. The linearity panels consisted of EDTA plasma or upper respiratory swab specimen matrix at 10-fold dilutions from 7.0 to 3.0 log_10_ IU/mL. Four replicates were tested at each of the 7.0 and 6.0 log_10_ IU/mL, whereas 8 replicates were tested at each of the remaining levels, except for the respiratory swab specimen matrix, where only 6 replicates were tested at 2.0 log_10_ IU/mL. The linearity panel replicates were tested on a single day.

### Clinical specimens for method comparison.

This study utilized 122 EDTA plasma specimens submitted to the Stanford Health Care Clinical Virology Laboratory between October 2016 and April 2021 for quantitative HAdV testing. All specimens were stored at −80°C prior to testing.

### Reference adenovirus qPCR.

Total nucleic acids were purified using 400 μL of EDTA plasma using the EZ1 virus minikit version 2.0 on the EZ1 Advanced XL instrument (Qiagen) and eluted in 60 μL of buffer AVE. HAdV DNA was quantitated using a laboratory-developed real-time PCR assay modified from Huang et al. (12). The assay was performed using the QuantiFast Pathogen +IC kit on the Rotor-Gene Q instrument (Qiagen). Each reaction used 10 μL of eluate, with a final reaction volume of 25 μL. Primer and probes (both from Biosearch Technologies) targeting the hexon and penton genes were as described previously but with black hole quencher 1 (BHQ-1) used as quencher rather than TAMRA (6-carboxytetramethylrhodamine). HAdV A and HAdV F primers were added at final concentrations of 400 nM, whereas primers HAdV B, HAdV C, and HAdV E were added at final concentrations of 280 nM. Final concentrations of probes were 100 nM for HAdV A and HAdV F probes, 64 nM for HAdV B and HAdV C probes, and 72 nM for HAdV E probes. Cycling conditions were as follows: hold at 95°C for 5 min, followed by 45 cycles of 95°C for 15 s and 60°C for 30 s. Detection was performed in the green (HAdV) and yellow (IC) channels; the threshold was set at 0.04 for the green channel and 0.07 for the yellow channel. The assay was calibrated using the Acrometrix adenovirus plasma panel (Thermo Fisher). The assay is linear from 2.3 to 6.8 log_10_ copies/mL.

### Statistical analysis.

The lower limit of detection was calculated using probit in R. Precision was also analyzed in R, using a custom script implementing the formula described in Chesher (13). Linearity was evaluated using ordinary least-squares regression in Prism version 6.0g (GraphPad, La Jolla, CA). Correlation and Bland-Altman plots were also generated in Prism. Positive percent agreement (PPA) and negative percent agreement (NPA) were calculated using the MedCalc Diagnostic test evaluation calculator (https://www.medcalc.org/calc/diagnostic_test.php, version 20.027). Kappa statistics were determined using www.graphpad.com/quickcalcs/kappa2/. Passing-Bablok regression analyses were performed in R using the mcr package (https://cran.r-project.org/web/packages/mcr/index.html).
